# Partially intraosseous schwannoma of the distal humerus with increased enhancement after biopsy: Radiologic-pathologic correlation^☆^

**DOI:** 10.1016/j.radcr.2021.12.050

**Published:** 2022-02-04

**Authors:** Shan S. Hansra, Colin N. Brown, Lisa H. Kang, Kurt B. Schaberg, Steven W. Thorpe, Dillon C. Chen

**Affiliations:** a Department of Radiology, University of California Davis Medical Center, 4860 Y Street, Suite 3100, Sacramento, CA 95817, USA; b Department of Pathology, University of California Davis Medical Center, 4400 V Street, Sacramento, CA 95817, USA; c Department of Orthopedic Surgery, University of California Davis Medical Center, 4860 Y Street, Suite 3100, Sacramento, CA 95817, USA

**Keywords:** Intraosseous schwannoma, Post-biopsy enhancement

## Abstract

Intraosseous schwannomas are rare benign tumors that most often occur at the mandible or sacrum. We present an unusual case of a bilobed schwannoma of the distal humerus with both intraosseous and extraosseous components. The extraosseous component was non-enhancing on initial MRI and enhanced on a subsequent MRI obtained after biopsy. We hypothesize that this change was attributable to decreased intra-tumoral pressure secondary to biopsy-related disruption of the tumor capsule.

## Background

Schwannoma is the most common peripheral nerve sheath tumor. It is composed of Schwann cells which form the myelin sheath of peripheral nerves. Masson coined the term “schwannoma” in 1923 to describe peripheral nerve sheath tumors distinct from neuromas [1].

Most schwannomas are solitary and sporadic, although approximately 5% occur in the setting of syndromes such as neurofibromatosis type 2 or schwannomatosis, often as multiple lesions [2]. Loss of function of the NF2 gene, a tumor suppressor gene that encodes the protein merlin, is implicated in the pathophysiology of most schwannomas [3,4]. Most peripheral nerve sheath tumors are benign [5,6]. About 50% of the cases of malignant transformation are associated with NF1 [7].

As peripheral nerve sheath tumors grow along the length of a nerve, they are typically fusiform [8]. Schwannomas are typically eccentrically located in relation to the parent nerve, with the nerve fibers draped over the tumor. In contrast, neurofibromas arise more centrally and expand the parent nerve [9]. When circumferential growth of a peripheral nerve sheath tumor is restricted by a bony canal or foramen, as occurs with tumors arising from spinal nerves at the neural foramina, the lesion may assume a dumbbell-shaped morphology [10,11]. While schwannomas typically occur in the soft tissues, bone involvement can occur in three ways: 1) tumor origin in the medullary cavity, 2) tumor arising within a nutrient canal, or 3) intraosseous extension of an extraosseous tumor [13].

Intraosseous schwannomas are very rare, accounting for 0.1%-0.2% of primary bone tumors [12,13]. A study involving 17 cases of primary bone schwannomas demonstrated a slight female predilection and a mean group age of 35 years. The most common complaint was pain rather than sensorimotor impairment [12]. The mandible and sacrum are the bones most commonly affected. This is hypothesized to be due to the long intraosseous course of the mandibular nerve and the abundance of sensorimotor nerves passing though the sacrum, respectively [13,14]. Others argue that schwannomas are simply more common in the head and neck and that sacral schwannomas should not truly be considered intraosseous [12,15]. Approximately 25% of intraosseous schwannomas occur in long bones [14]. A likely contributing factor to the rarity of intraosseous schwannomas of long bones is the scarcity of myelinated nerves within these bones. While the sensory nerves of the periosteum are myelinated, intraosseous nerves are largely small, unmyelinated vasomotor nerves [16]. The first intraosseous schwannoma of the humerus was described in 1939 by Gross and colleagues. To our knowledge, a total of five humeral intraosseous schwannomas have been reported in the literature thus far [17], [18], [19], [20], [21].

At gross pathology, schwannomas have a capsule derived from epineurium and are often seen growing along a parent nerve [22,23]. At microscopy, schwannomas demonstrate two major patterns. Antoni A pattern tissue is highly cellular and composed of interlacing spindle cells. Antoni B pattern tissue is hypocellular with loose, myxoid stroma. Verocay bodies are a characteristic histologic finding in schwannomas, present in areas of Antoni A pattern tissue, and consist of two palisading bands of nuclei with an interposed anuclear zone [24]. “Ancient” schwannomas are those in which advanced degenerative changes such as cysts, hemorrhage, or calcification are present [25]. At immunohistochemistry, both schwannomas and neurofibromas typically demonstrate strong S100 positivity and can display scattered CD34 expression [26]. While schwannomas are comprised almost entirely of Schwann cells, neurofibromas typically demonstrate intra-tumoral axons on neurofilament staining [24].

On radiographs, intraosseous schwannomas have benign imaging features such as a narrow zone of transition and little to no periosteal reaction [12]. However, these features are nonspecific [27].

On MRI, schwannomas are typically well-defined solitary masses that have a fusiform appearance and can often be seen growing eccentrically along a parent nerve. A “split-fat” sign can be present, visible as a thin rim of fat surrounding the tumor, often most apparent at the ends of the lesion on long-axis images. However, this sign is not specific for schwannoma and can be seen in other soft tissue tumors such as myxomas and myxofibrosarcomas [28]. Schwannomas are isointense-to-hypointense compared to muscle on T1-weighted images, hyperintense on T2-weighted images, and enhance after contrast administration. Differentiating between a schwannoma and a solitary neurofibroma is difficult based on imaging alone [29]. Also difficult is distinguishing schwannomas from malignant peripheral nerve sheath tumors. However, features concerning for malignancy include size greater than 5 cm and infiltrative tumor margins [30].

## Case report

A 17-year-old woman presented with a painless mass in the distal right upper arm which she had noticed one week earlier. She denied prior trauma and had no significant past medical history. Physical examination was notable for a firm and immobile mass at the anteromedial distal upper arm. Laboratory evaluation inclusive of complete blood count, basic metabolic panel, and inflammatory markers was normal.

**Fig. 1:**
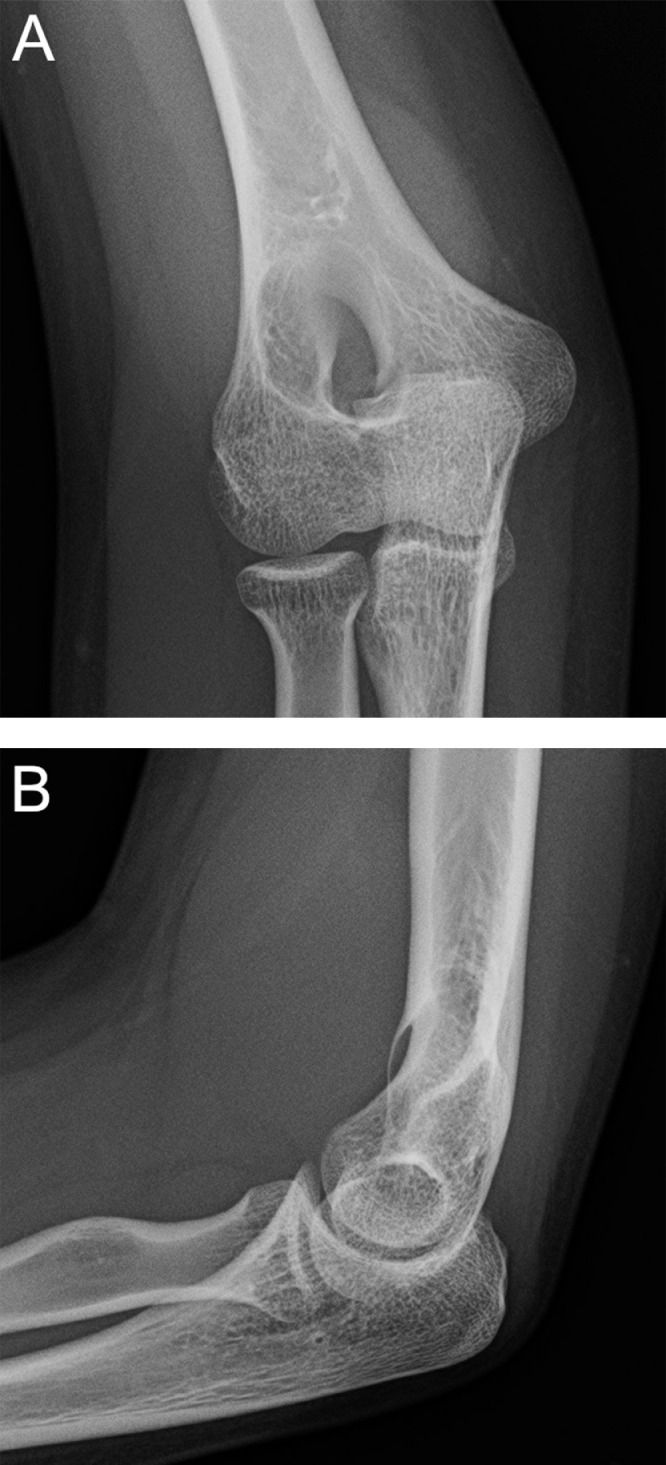
AP and lateral projections of the right elbow showing a soft tissue mass involving the distal humerus (A) and a lucent lesion with a thin sclerotic rim at the anterior aspect of the distal humerus (B).

Radiographs demonstrated a soft tissue mass anterior to the distal humerus (Fig. 1A) in addition to a well-defined lucent bone lesion with a thin sclerotic rim located centrally at the distal humeral metaphysis (Fig. 1B).

**Fig. 2:**
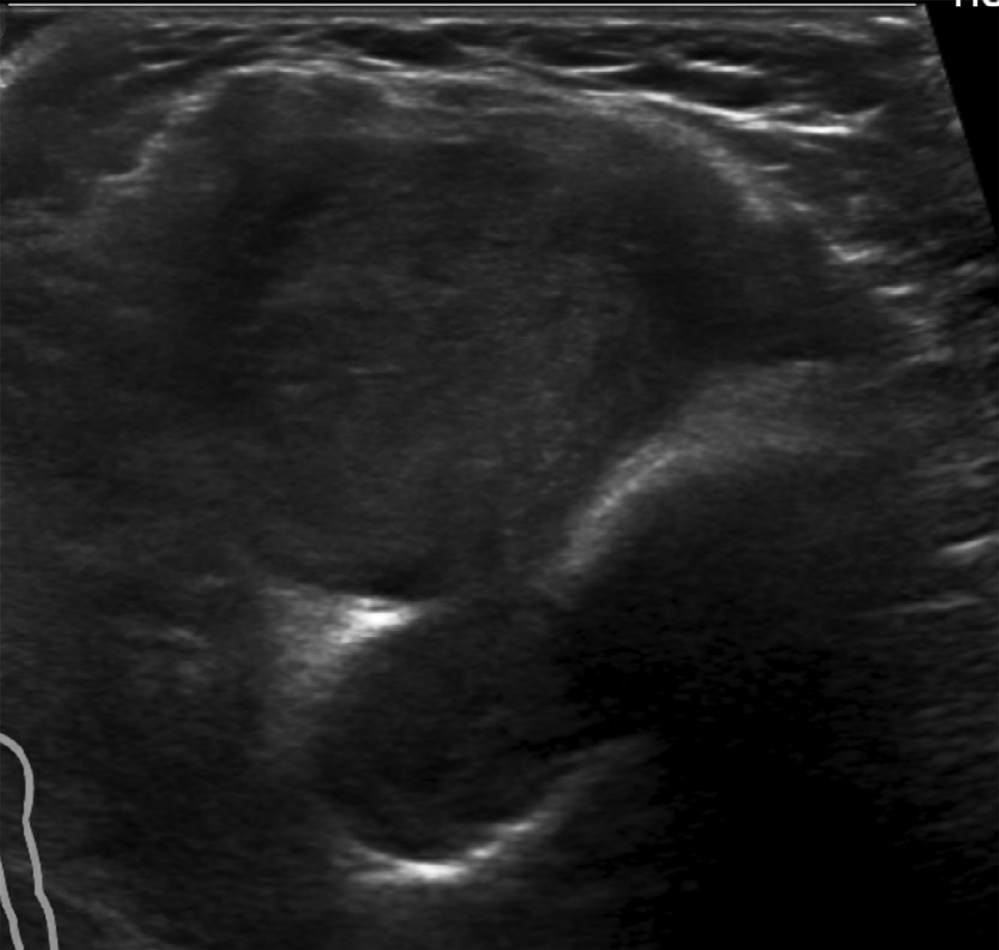
Transversely oriented ultrasound of the right antecubital region demonstrating a soft tissue mass involving the anterior aspect of the humerus. The center of the mass is isoechoic, and the periphery is hypoechoic.

Sonography was subsequently performed and showed a deep soft tissue mass with circumscribed margins, peripheral decreased echogenicity, and somewhat greater echogenicity centrally (Fig. 2). There was minimal vascularity within the lesion on color Doppler evaluation.

**Fig. 3:**
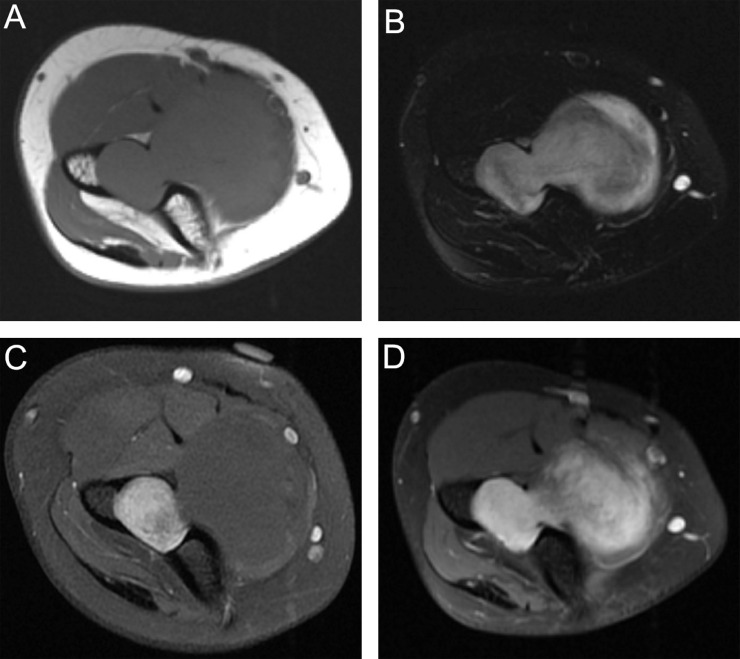
A. Axial T1W images demonstrate the intraosseous and extraosseous components of the mass which are isointense and joined through a defect in the anterior distal humerus.;B Axial T2W fat-suppression images show both components are hyperintense with the periphery of the extraosseous component having increased signal compared to the center.;C.Axial T1W fat-suppression post-contrast pre-biopsy images show the intraosseous component enhances while the extraosseous component does not.;D. Axial T1W fat-suppression post-contrast post-biopsy images demonstrates new interval enhancement of center of the extraosseous component with persistent non-enhancement of the outer rim.

**Fig. 4:**
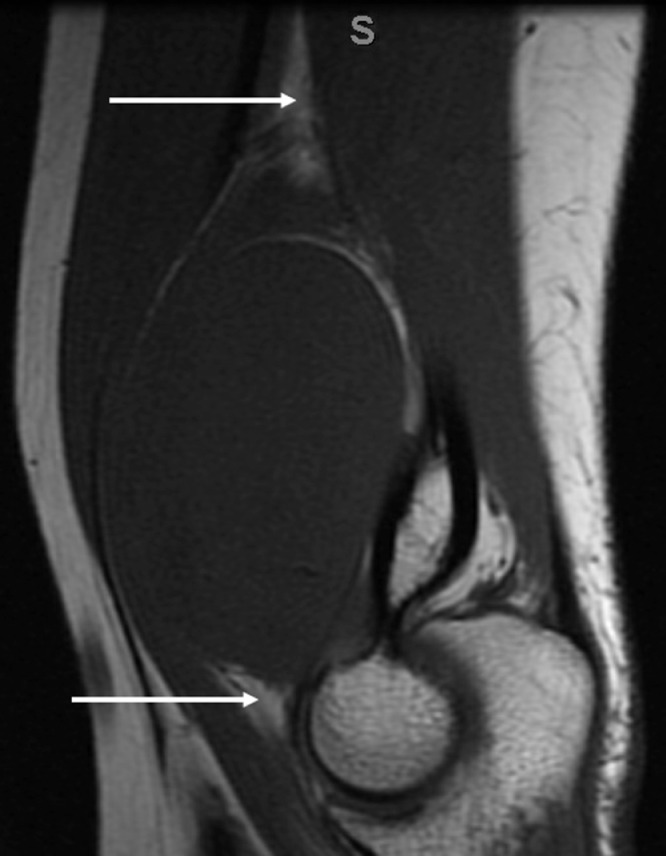
Sagittal T1W image demonstrates the “split-fat” sign – tapered fatty rinds on either end of an intramuscular mass.

MRI was then performed, demonstrating a dumbbell-shaped mass with an intraosseous component measuring up to 2.5 cm located centrally in the distal humeral metaphysis and a larger extraosseous component extending anteromedially through a cortical defect and measuring up to 5.0 cm. The mass was isointense to muscle on T1-weighted images. On T2-weighted images, the mass was hyperintense with mild heterogeneity and higher signal intensity at the periphery of the extraosseous component (Figs. 3A and B). A split-fat sign was present at the proximal and distal aspects of the extraosseous component (Fig. 4). Following intravenous contrast administration (gadoteridol, Bracco Diagnostics, Monroe Township, NJ), the intraosseous component enhanced avidly and the extraosseous component was non-enhancing (Fig. 3C).

Ultrasound-guided fine needle aspiration and core biopsy of the mass was then performed, with samples obtained from both the enhancing intraosseous component and the non-enhancing extraosseous component.

Histologic specimens of both tumor components showed a proliferation of spindle cells with areas of variable cellularity and no evidence of malignancy. Verocay bodies, although poorly formed, were identified. Immunohistochemistry demonstrated diffuse, strong S100 positivity and scattered CD34 positivity, but neurofilament staining was negative for intra-tumoral axons. Based on these features, the biopsy was interpreted as consistent with benign peripheral nerve sheath tumor, favoring schwannoma with neurofibroma not entirely excluded.

After discussion of the biopsy results, the patient elected observation with MRI follow-up. A repeat MRI was performed approximately 3 months after the initial MRI and biopsy. On this second MRI, the extraosseous component of the lesion enhanced heterogeneously (Fig. 3D); findings were otherwise similar to those on the initial MRI.

**Fig. 5:**
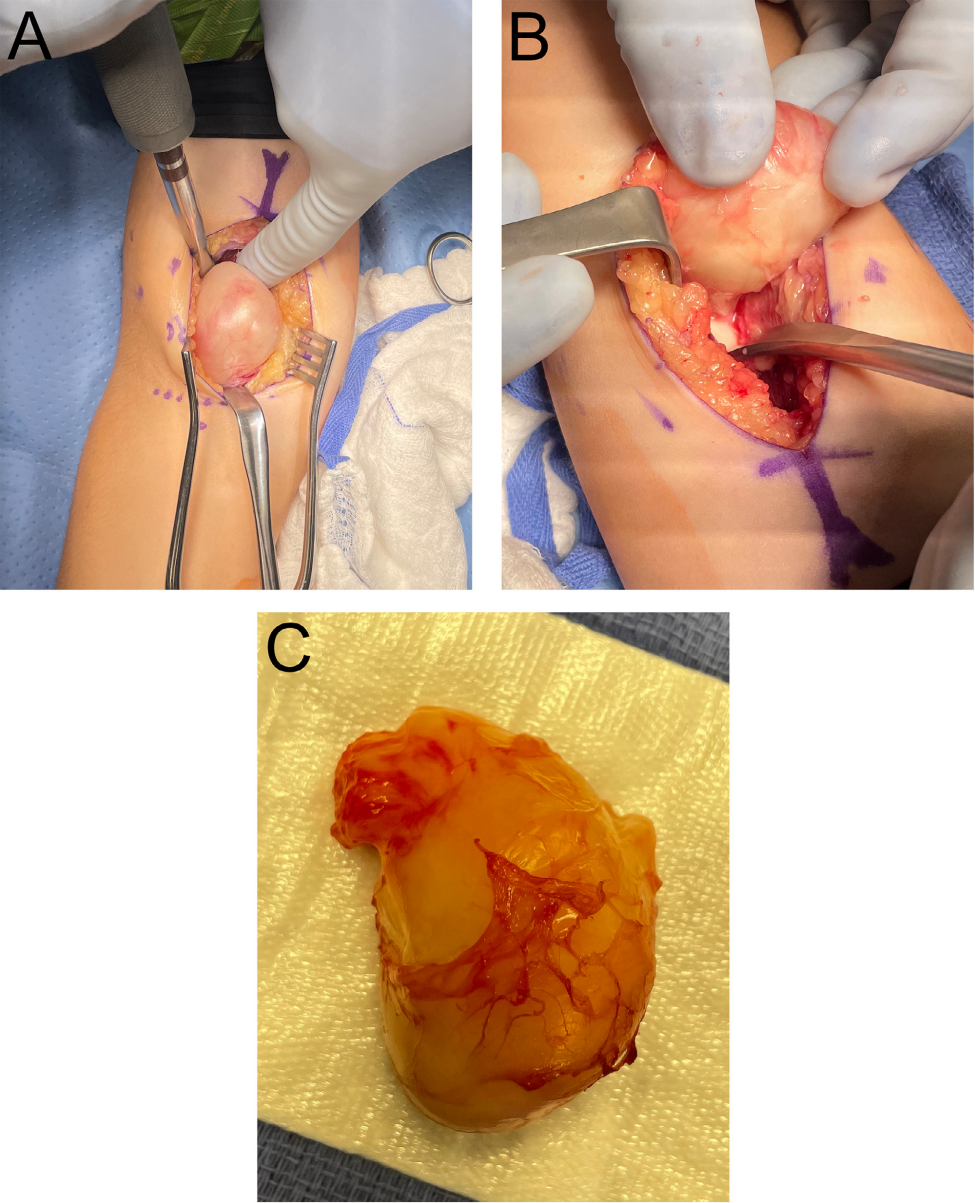
A. Visualization of the extraosseous mass after dissection through the antecubital fascia and brachialis muscle.;B. Lifting the extraosseous mass to show its connection with the intraosseous component.; C. The excised extraosseous mass.

About four and a half months after the initial MRI, the patient decided to proceed with surgical excision. The extraosseous component was freed after careful dissection (Figs. 5A, B, and C). The intraosseous component was then removed by curette and the bony void was filled with allograft and synthetic bone putty.

**Fig. 6:**
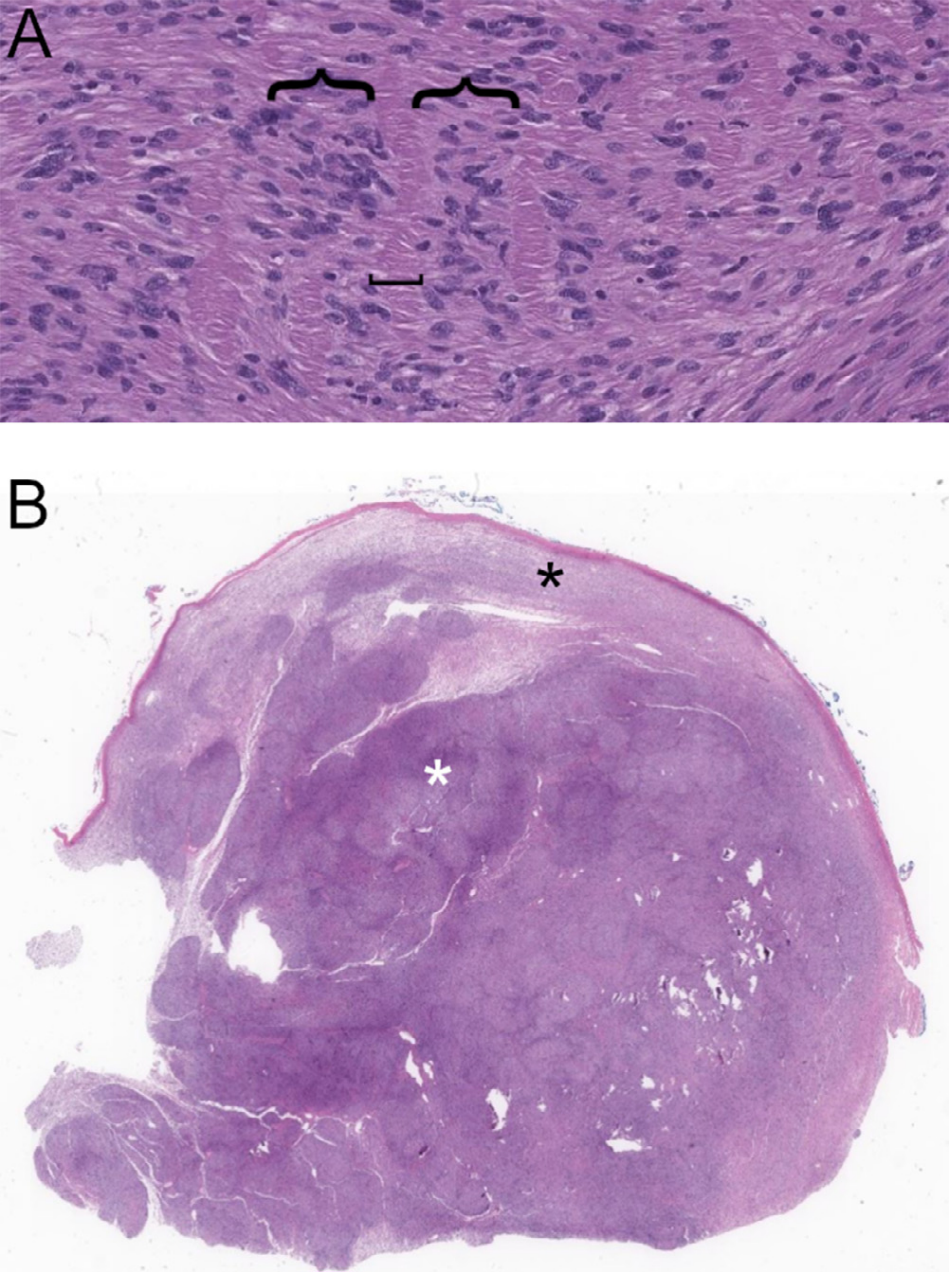
A. 10X magnification through the extraosseous component demonstrates Verocay bodies consisting of two palisading rows of cells (denoted with {) with an anuclear zone in between (denoted with [).; B.1X magnification of the extraosseous component illustrates a relatively cellular center (Antoni A, white *) and a hypocellular rim (Antoni B, black *). A discontinuous pseudocapsule is seen surrounding the mass.

Histopathology of the surgical specimen demonstrated the presence of Verocay bodies, confirming the diagnosis of schwannoma (Fig. 6A). The extraosseous mass consisted of cellular Antoni A pattern tissue centrally and hypocellular, myxoid Antoni B pattern tissue at the periphery. Discontinuity of the tumor capsule was noted (Fig. 6B).

Post-operatively, the patient experienced hyperesthesia in the median nerve distribution which improved over time.

## Discussion

Of the three mechanisms previously described for osseous involvement by schwannomas, it seems most probable that the tumor in our case originated within a nutrient canal, with subsequent extra-osseous growth producing the dumbbell-shaped morphology. While the largest nutrient foramen of the humerus is located at the diaphysis, smaller nutrient foramina are present at the distal humerus [16,31].

The imaging appearance of the mass in the current case parallels the histopathologic findings, with the relatively T2-hyperintense and hypoenhancing periphery of the extraosseous component corresponding to areas of hypocellular Antoni B pattern tissue, and the less T2-hyperintense, and enhancing central region corresponding to cellular Antoni A pattern tissue (Figs. 2 and 3).

An unusual feature of this case is the lack of enhancement of the extraosseous component on the initial MRI and avid enhancement of the same portion of the tumor on the subsequent MRI. As the initial biopsy specimen and resection specimen were histologically similar and there was no other intervention between the two MRIs, we hypothesize that this change may have occurred due to a decrease of intra-tumoral pressure secondary to disruption of the tumor pseudocapsule by the biopsy. This theory is supported by the presence of capsular disruption noted at gross examination of the resection specimen. We estimate the scan delays to be similar for both MRIs. Furthermore, as there was no change in enhancement over the duration of the contrast-enhanced potions within either the initial or subsequent MRI, difference in scan delay is unlikely to account for difference in tumor enhancement.

A similar phenomenon of increased post-biopsy enhancement has previously been reported in intramuscular myxomas by Wang et al. [32]. They attributed this effect to the disruption of the tumor pseudocapsule, resulting in decreased intra-tumoral pressure and increased tumor uptake of contrast. We suspect that the unusual enhancement pattern in our schwannoma case can similarly be explained by this hypothesis.

The concept of increased interstitial pressure in tumors impeding molecular transport was described by Jain in 1987 [33]. Tumors are known to express high levels of cytokines and growth factors such as vascular endothelial growth factor, which lead to highly permeable and irregular vascular networks. Such “leaky” vessels result in the accumulation of fluid in the interstitial tissue, thus increasing interstitial pressure. Various methods of decreasing interstitial pressure have been studied in order to increase uptake of therapeutic agents into the tumor. These include such non-physical methods as angiogenesis inhibitors and physical methods as radiation and hyperthermia [34], [35], [36], [37].

In conclusion, we present a rare case of intraosseous schwannoma of the distal humerus, likely arising within a nutrient canal. The histopathologic features noted after resection parallel the imaging findings, which in turn reflect the variable composition and cellularity of schwannomas. We hypothesize that increased enhancement of the extraosseous component following biopsy was secondary to disruption of the tumor capsule and associated decrease of intra-tumoral pressure. Although these lesions are rare, radiologist awareness of intraosseous schwannoma can facilitate efficient diagnosis and appropriate treatment of these benign tumors.

## Patient consent

Written informed consent was obtained from the patient's parents.
